# Antioxidant Activity and Cytotoxicity Effect of Cocoa Beans Subjected to Different Processing Conditions in Human Lung Carcinoma Cells

**DOI:** 10.1155/2016/7428515

**Published:** 2016-03-13

**Authors:** Deborah Bauer, Joel Pimentel de Abreu, Hilana Salete Silva Oliveira, Aristoteles Goes-Neto, Maria Gabriela Bello Koblitz, Anderson Junger Teodoro

**Affiliations:** ^1^Nutritional Biochemistry Core, Federal University of Rio de Janeiro State, Avenida Pasteur 296-Urca, 22290-240 Rio de Janeiro, RJ, Brazil; ^2^Graduate Program in Biotechnology, State University of Feira de Santana, Avenida Transnordestina, S/N, Novo Horizonte, 44036-900 Feira de Santana, BA, Brazil; ^3^Food and Nutrition Graduate Program, Nutritional Biochemistry Core, Federal University of Rio de Janeiro State, Avenida Pasteur 296-Urca, 22290-240 Rio de Janeiro, RJ, Brazil

## Abstract

Lung cancer is a common malignancy in men and the second leading cause of cancer-related mortality in men in the western world. Phenolic cocoa ingredients have a strong antioxidative activity and the potential to have a protective effect against cancer. In the present study, we have evaluated the influence of cocoa beans subjected to different processing conditions on cell viability and apoptosis of human lung cancer cells (A549). We measured the viability of lung cells treated with cocoa beans, unroasted slates (US), roasted slates (RS), unroasted well fermented (UWF) cocoa, and roasted well fermented (RWF) cocoa for 24 h. Using an MTT assay, we observed a decrease in the viability of A549 cells after treatment with cocoa bean extracts. Flow cytometer analysis revealed that cocoa beans increased the percentage of cells in sub-G_1_ phase and promoted up to twofold increase of apoptotic cells when compared to the control group. Taken together, the present study suggests that cocoa beans may have a protective effect against lung cancer.

## 1. Introduction

Lung cancer is a major health concern since it is one of the leading causes of death worldwide [1, 2]. It is estimated that the annual incidence rate is nearly 1.23 million. In Brazil, it is the second most frequent type of cancer and it has the highest mortality rate, due to late diagnosis and the aggressiveness of the tumor type. In 80–90% of cases, it is caused by smoking and exposure to pollutants. The existing therapeutic strategies for cell lung cancer include surgery, radiotherapy, chemotherapy, and physical therapy. The survival rate of non-small-cell lung cancer patients is less than 1% [3, 4].

The proposals that cancer might be preventable, and that food and nutrition might influence the risk of cancer, were first made in the 19th and 20th centuries. Throughout recorded history, wise choices of food and drink, and of habitual behavior, have been recommended as a protective measure against cancer [5]. Researchers are still unsure about the role of diet in lung cancer. Bright yellow-orange beta-carotene is one of a number of carotenoids thought to have anticancer activity even greater than vitamin A. Other possible lung protectors are foods high in bioactive compounds such as vitamin C and other antioxidants present in fruits and vegetables. These nutrients may protect lung linings but cannot totally prevent damage [6].

Among the different bioactive compounds, phenolic compounds from fruits and vegetables have gained much attention over the years because of their antioxidant activity that indirectly reflects their potential effects on human health [7, 8]. Some studies reported that phenolic compounds found in cocoa beans may present different properties such as antioxidant, anticarcinogenic, and antiradical activities [9–11]. Polyphenols are the main antioxidant-active constituents of cocoa. Flavanols and procyanidins have previously been identified as the active antioxidant agents of cocoa [12]. The polyphenol content of cocoa products depends on many factors, especially the cultivated variety and the postharvest handling that includes fermentation, drying, and roasting of the beans and nibs. There is evidence that fermentation and roasting of the beans tend to reduce their flavanol content [13, 14]. The antioxidant activity of the phenolic compounds is primarily due to their redox properties that allow them to act as reducing agents, hydrogen donors, and scavengers of reactive oxygen species (ROS) and metal ions [8, 15–17]. The emergence of natural extracts with antioxidant properties may help reduce the current dependence on synthetic drugs.

Potential mechanisms for cancer prevention of bioactive compounds include prevention of DNA adduct formation enhanced carcinogen elimination, inhibition of inflammatory processes, and a direct cytotoxic effect on tumour cells [18–20]. In line with efforts to balance the conservation of biodiversity and encourage the controlled exploitation of plant resources for economic gain, especially in biopharming, waste of valuable resources should be minimized [17]. The aim of this study was to evaluate and compare the antioxidant activity of cocoa beans classified as slate, roasted, and unroasted with well fermented beans submitted to the same processing and their cytotoxic effects on human lung carcinoma cell line (A549).

## 2. Methods

### 2.1. Samples and Extractions

Samples of cocoa beans, unroasted slates (US), roasted slates (RS), unroasted well fermented (UWF) cocoa, and roasted well fermented (RWF) cocoa were harvested and preprocessed (fermented and dried) in the cocoa producing region of Ilhéus (Bahia, Brazil). These samples were classified according to their fermentation status and donated by a company of the cocoa sector from the same region. At least three different lots of each cocoa class were mixed to form the samples used in this study. The roasting as well as the fine grinding of all beans was carried out in the State University of Feira de Santana (BA). All samples were kept at −5°C and sent to the Functional Foods and Biotechnology Laboratory of the Federal University of Rio de Janeiro State (UNIRIO), where all analyses were conducted.

### 2.2. Extraction of Samples

The samples of cocoa were extracted with 3 different solution extractors: methanol (I), methanol 50% (II), and methanol 50% : acetone 70% (1 : 1) (III). 1.25 g of sample was weighed and suspended in 10 mL of extracting solution for 1 hour under stirring, protected from light. The crude extracts were filtered and completed in 25 mL with distilled water. For cellular analysis, 1.25 g of each sample was weighed and suspended in saline solution (PBS) at 2% dimethyl sulfoxide (DMSO).

### 2.3. Total Phenolic Assay

Total phenolic content of the extracts was determined according to the Folin-Ciocalteu method as described by Singleton and Rossi [21] with minor modifications. Aliquots of 0.5 mL of the extracts were added to 2.5 mL of Folin-Ciocalteu reagent and 2.0 mL of 4% sodium carbonate solution and the mixture was allowed to rest for 2 hours in the dark. Measurements were performed at 750 nm in triplicates, applying a Turner® 340 spectrophotometer. Gallic acid, in the concentration range of 0–100 mg/mL^−1^, was used to construct a calibration curve. The concentration of total phenolic compounds in the extract was expressed as gallic acid equivalents, which reflect the phenolic content as the amount of gallic acid in mg/100 g dry weight of the samples.

### 2.4. Antioxidant Activity Analyses

#### 2.4.1. DPPH Assay

Aliquots of 0.5 mL of the extracts were mixed with 2.5 mL DPPH methanolic solution (0.06 mM) and allowed to react for 1 hour, in the dark. Measurements were performed at 515 nm applying a Turner 340 spectrophotometer. The analysis was performed in triplicates; the decline in the DPPH radical absorbance concentration caused by the extracts was compared to a trolox standard. The results were expressed as *μ*mol trolox equivalents/g dry basis. [22].

#### 2.4.2. Trolox Equivalent Antioxidant Capacity (ABTS/TEAC)

The TEAC^•+^ cation was prepared by mixing a TEAC stock solution (7 mM in water) with 2.45 mM potassium persulfate. This mixture was allowed to stand for 16 hours at room temperature until the reaction was completed and the absorbance was stable.

The antioxidant capacity assay was carried out by the improved ABTS/TEAC method as described by Rufino et al. [23]. TEAC solution (2.5 mL) was added to extracts or commercial antioxidant (trolox) and mixed thoroughly. Absorbance was recorded at 734 nm during 6 min. Aliquots of 5, 10, and 20 *μ*L of the extracts were tested and their volume was completed to 0.5 mL with water. Results were expressed as *μ*mol trolox/g dry basis.

#### 2.4.3. Ferric Reducing Ability (FRAP)

The extracts were measured for antioxidant activity by FRAP according to Rufino et al. [24]. Aliquots of 2.7 mL of TPTZ reagent (ferric 2,4,6-tripyridyl-s-triazine) were mixed with 0.5 mL of sample extract (aliquots 5, 10, and 20 *μ*L). After 30 min at 37°C temperature, the absorbance was read at 595 nm. The antioxidant capacity (FRAP) was expressed as Fe^3+^ equivalents (*μ*mol Fe^3+^/g dry basis).

#### 2.4.4. Cell Culture and Treatment Protocol

Cell lines were obtained from the Rio de Janeiro Cell Bank which certified their identity and quality (INMETRO, Rio de Janeiro, RJ, Brazil). Human lung carcinoma cell line (A549) was plated in 25 cm^2^ tissue culture flasks (5.0 × 10^6^ cells/flask) and maintained routinely in Dulbecco's Modified Eagle's Medium-high glucose (DMEM) supplemented with 10% Fetal Bovine Serum (FBS) and 1% Penicillin (PS), pH 7.4, under 5% CO_2_ atmosphere. Stock flasks were grown to 70% confluence and subcultured routinely. Medium renewal was done 3 times weekly. For each experiment, cells were seeded at 3.5 × 10^5^ cells/cm^2^ and 2 × 10^4^ cells/cm^2^ densities in 6-well plates and 96-well plates for cell cycle and cell proliferation analyses, respectively. After 24 h, medium was removed and cells were treated with increasing concentrations of cocoa nibs extract (100 to 10000 *μ*g/mL) dissolved in DMEM. The controls, DMEM and DMEM + 2% DMSO, were included on each plate. The cells were then incubated for 48 hours.

#### 2.4.5. Cell Viability

Cell viability was monitored by MTT assay (Amresco, Solon, OH). MTT (3-(4,5-dimethylthiazol-2-yl)-2,5-diphenyltetrazolium bromide) is a pale yellow substrate that is reduced by living cells to yield a dark blue formazan product. This requires active mitochondria, and even recently dead cells do not reduce significant amounts of MTT. Exponentially growing cells were adjusted to 2.0 × 10^4^/cm^2^ with DMEM, plated in 96-well plates (Corning, Tewksbury, MA) at 200 *μ*L/well and incubated for 24 h according to the routine procedure. The cells were then incubated with cocoa nibs unroasted slates (US), roasted slates (RS), unroasted well fermented (UWF) cocoa, and roasted well fermented (RWF) cocoa (5–10 mg/mL) for 48 h (6 wells for each sample). Each well was also incubated with MTT (10 *μ*L/well; 5 g/mL) for 4 h. After 85 *μ*L/well the liquid was removed and 50 *μ*L/well sodium dodecyl sulfate was added to dissolve the solid residue. Finally, the absorbance was measured using a microplate reader (POLARIS, CELER®) at 570 nm. The cell proliferation inhibition rate (CPIR) was calculated using the following formula: CPIR = (1 − average value of experimental group/average value of control group) × 100%.

#### 2.4.6. Cell Cycle Analysis

Cells were rinsed briefly with calcium and magnesium-free phosphate-buffered saline and detached with trypsin at room temperature. After centrifugation, the cells were washed twice with phosphate-buffered saline; cells were resuspended in 500 *μ*L of ice-cold Vindelov solution [25] containing 0.1% Triton X-100, 0.1% citrate buffer, 0.1 mg/mL RNase, and 50 mg/mL propidium iodide (Sigma Chemical Co., St. Louis, MO). After 15 min of incubation, cell suspension was analysed for DNA content by flow cytometry using a FACSCalibur flow cytometer (Becton Dickinson, Mountain View, CA). The relative proportions of cells with DNA content indicative of apoptosis (<2n), G_0_/G_1_ diploid (2n), S (phase >2n but <4n), and G_2_/M phase (4n) were obtained and analyzed using the CellQuest WinMDI 2.9. The percentage of cell population at a particular phase was estimated with FlowJo software. Cell dissociation procedure does not affect fluorescence under the experimental conditions that were used in this study or in any other studies of which we are aware. Nuclei of viable cells were gated according to FL-2W × FL2-A relation.

#### 2.4.7. Apoptosis Assay

To measure the rate of apoptosis, the cells were subjected to staining with Annexin V conjugated to FITC (BD Pharmingen, San Diego, CA). The nonadherent cells were collected, and adherent cells were quickly washed with buffered saline solution (BSS) calcium/magnesium-free and were detached with trypsin/EDTA 0.125% (Sigma chemical Co., St. Louis, USA) at room temperature. Subsequently, apoptotic and necrotic cells were stained with Annexin VFITC/propidium iodide (PI) (BD Pharmingen, New Jersey, USA) according to the manufacturer's instructions, quantified by flow cytometer (FACSCalibur, BD Bioscience, New Jersey, USA), and analyzed using two specific programs, Cell Quest and FlowJo software.

### 2.5. Statistical Analysis

Results are presented as mean with the corresponding standard deviation of 3 independent experiments done in triplicates (*n* = 9). Data were analysed with the statistical software GraphPad Prism (version 5.04, GraphPad Software, San Diego, CA) and Statistica (version 7.0, StatSoft Inc., Tulsa, OK). One-way analysis of variance (ANOVA) test with the posttest of Tukey at a confidence level of 95% was used to test cell viability, cell cycle, and apoptosis.

## 3. Results and Discussion

### 3.1. Phenolic Compounds and Antioxidant Activity of Cocoa Beans Samples

**Figure 1:**
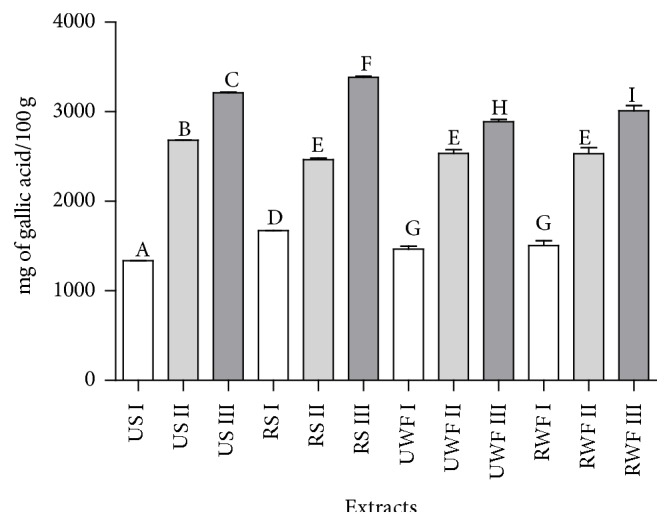
Total phenolic compounds of cocoa nibs unroasted slates (US), roasted slates (RS), unroasted well fermented (UWF) cocoa, and roasted well fermented (RWF) cocoa. Extracting solutions: I, methanol; II, methanol 50%; III, 1/2  50% methanol : 1/2  70% acetone. Means with different letters differ significantly (*p* < 0.05, Tukey's test).

The method that yielded higher phenolic extracts was the one using acetone, in all samples tested, as can be observed in Figure 1. Dreosti [26] reported that 60% of the total phenolics in raw cocoa beans are flavanol monomers (epicatechin and catechin) and procyanidin oligomers (dimer to decamer) [27]. Benayad et al. [28], Cheng et al. [29], and Boulekbache-Makhlouf et al. [30] have shown that the use of acetone, when compared to the use of other polar organic compounds, potentiated the extraction of flavonoids and flavonols from different plant materials. In addition, extraction of procyanidins from cocoa with acetone solutions has been successfully accomplished at least since 1999.

The present work found amounts of phenolic substances (Table 1) compatible with the results displayed by Kadow et al. [31] for both raw and “fermentation-like” treated samples. Unprocessed cocoa beans usually present a high phenolic content of about 12–18% (dry weight) [32]. The processing of raw cocoa includes a number of stages and each stage in the processing alters cocoa's chemistry and composition [11]. When extractor III was considered, there were significant differences among all samples tested and slates showed higher phenolic content when compared to well fermented samples. Generally, it is expected that well fermented beans show lower phenolic contents, because low mass phenols are related to astringency and anthocyanins are related to the purple color of unfermented beans, both considered undesirable characteristics in chocolate [33, 34].

**Figure 2:**
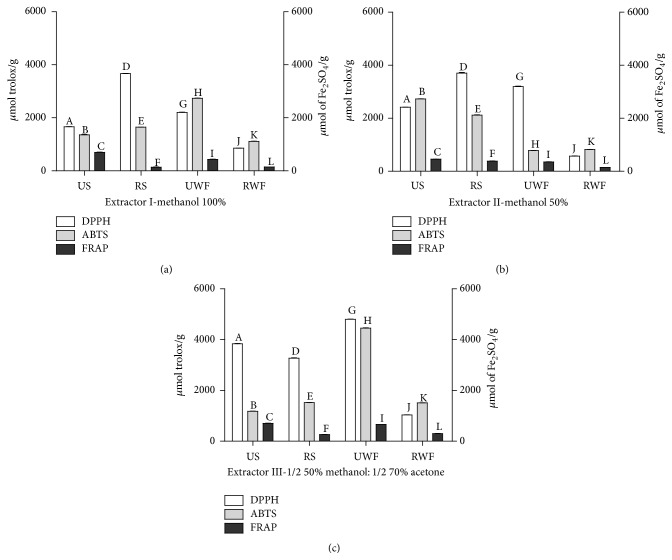
Antioxidant activity of cocoa nibs unroasted slates (US), roasted slates (RS), unroasted well fermented (UWF) cocoa, and roasted well fermented (RWF) cocoa for DPPH, TEAC, and FRAP assays by different extracting solutions (I–III). Means with different letters differ significantly (*p* < 0.05, Tukey's test).

Figures 2(a)–2(c) clearly show that the overall higher values were obtained for the DPPH method, followed by TEAC and FRAP, respectively. The slate samples showed higher antioxidant activity compared to well fermented beans or nibs. All these findings were in agreement with previous results as it was expected to obtain higher antioxidant activity in extracts with higher concentrations of phenolic compounds [14, 35–37].

The roasted slate (RS) samples showed a decrease in antioxidant activity after fermentation. Roasting is considered one of the processing steps of the cocoa nibs that leads to the loss of phenolic compounds and should cause a decrease in the antioxidant activity, as may be seen when comparing UWF and RWF samples (Figure 2). It is possible that, during roasting, while phenolic compounds are degraded, other antioxidant potential compounds are formed through the Maillard reaction, especially reductones and melanoidins [38, 39]. The reason why this formation may have been significant in slates but not in well fermented beans is probably related to the different compositions of these two samples before the roasting process.

FRAP assay showed a decrease in antioxidant activity after roasting. According to Apak et al. [40], FRAP methodology is not capable of measuring the antioxidant activity of compounds in complex matrix, since it takes more time to perform the analysis of antioxidant function, and not all antioxidants have the specific ability to reduce iron [41].

Recovery of antioxidant compounds from plant materials is typically accomplished through different extraction techniques, taking into account their chemistry and uneven distribution in the plant matrix. These compounds tend to present different polarities as well as other variable characteristics. Thus, the solubility in a particular solvent is a unique feature of the phytochemicals to be taken into account. Methanol and solutions of 50% methanol in water are commonly applied solvents that efficiently extract phenolic compounds. Methanol and acetone are also suitable solvents for anthocyanin extraction from various raw materials [42–44], and acetone-water mixtures have been suggested to grant better extraction results of procyanidins and phenols when compared with other extractors [45].

It is now recognized that diet and nutrients play an import role in cancer development and progress, with many dietary components found to be associated with cancer risk. However, almost all the clinical intervention trials with isolated nutrients, such as vitamin A, vitamin E, vitamin C, and phenolic compounds supplements, failed to demonstrate their protective effects against cancer. Due to the complexity of cocoa matrix, it is very difficult to characterize all components and even say which major component is responsible for the cytotoxic effect, due to the synergistic and antagonistic effects.

### 3.2. Cells Results

#### 3.2.1. Effect of Cocoa Extracts on Cell Viability

**Figure 3:**
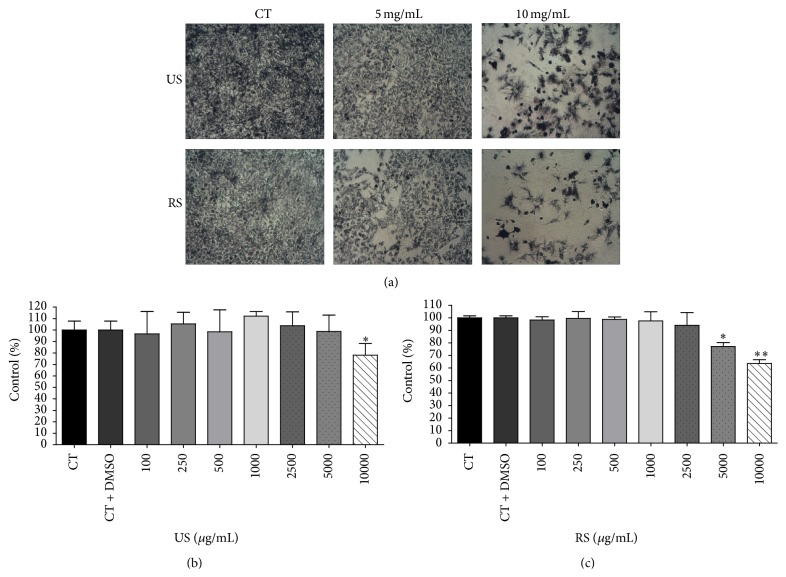
The effect of unroasted slates (US) and roasted slates (RS) nibs extract in cell control (a). Cocoa nibs US (b) and cocoa nibs RS (c), after forty-eight hours on viability A549 cells after exposure using MTT assays. The results are expressed as mean ± standard error and significant differences between cells treated with US and RS nibs extract (100–10000 *μ*g/mL) were compared using Tukey's test (^*∗*^ *p* < 0.05; ^*∗∗*^ *p* < 0.01).

The slate nibs were the supplement which caused the largest decrease in viability compared to control (34.45%, Figure 3), while cells exposed to US had the highest percentage of viability, 78.07%, at a concentration of 10 mg/mL (Figure 3(b)). However, surprisingly, crisp sample obtained higher potential reduction in cell viability (Figure 3(c)), with cell viability of 77.15% (5000 *μ*g/mL) and 63.55% (10.000 *μ*g/mL).

**Figure 4:**
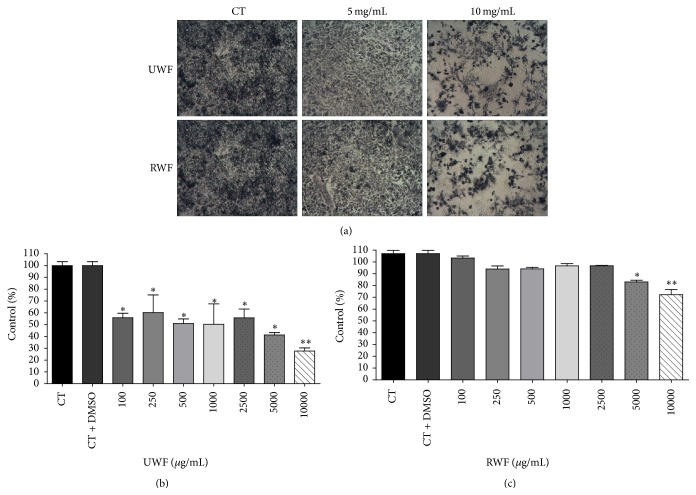
The effect of unroasted well fermented (UWF) nibs and roasted well fermented (RWF) nibs extracts in cell control (a). UWF cocoa nibs (b) and RWF cocoa nibs (c), after forty-eight hours on viability A549 cells after exposure using MTT assays. The experiment is expressed as mean ± standard error and significant differences between cells treated with UWF and RWF nibs extract (100–10000 *μ*g/mL) were compared using Tukey's test (^*∗*^ *p* < 0.05; ^*∗∗*^ *p* < 0.01).

Well fermented cocoa bean extract decreased the number of viable A549 cells within 48 hours. In UWF sample, cell viability decreased from the concentration of 100 *μ*g/mL by 45% compared with the control group (*p* < 0.05) (Figures 4(a) and 4(b)). The concentrations that caused the largest decrease in cell viability were 5000 and 10000 *μ*g/mL, reduced by 58.77% and 72.35%, respectively (Figure 3(b)). For RWF sample, the reduction was smaller (Figure 3(c)), with effect only at concentrations of 5000 (83.07% viable cells) and 10000 *μ*g/mL (72.20% viable cells).

Cell culture studies constitute a useful tool to elucidate the molecular mechanisms of action of cocoa extracts and their polyphenolic compounds in different cancer cell lines. It has been shown that cocoa components induced a time-dependent regulation of survival/proliferation pathways in HepG2 liver cells [46]. Moreover, a cocoa procyanidin fraction inhibited TPA-induced neoplastic transformation of JB6P+ mouse epidermal cells, COX-2 expression, and phosphorylation of MEK and p90 ribosomal s6 kinase and attenuated activator protein-1 (AP-1) and NF-*κ*B stimulations [47].

The physiological impact of polyphenols depends on their absorption. However, it is important to bear in mind that the most common polyphenols in diet are not necessarily the most bioavailable, since their structure plays an important role. Most native polyphenols in foods are in glycoside form (flavonols, flavones, flavanones, isoflavones, and anthocyanins), together with the less frequent oligomers (proanthocyanidins), which cannot be absorbed in the intestinal mucosa [48]. Through this, we can observe that the sample that most reduced the viability of human lung carcinoma cells was again the sample which had the parent compounds of the modified crude cocoa, such as well fermented sample, and also the sample which has not been roasted, since many bioactive compounds are lost during this processing.

#### 3.2.2. Effect of Cocoa Extracts on Cell Cycle Progression

Uncontrolled cell proliferation is a characteristic of cancer [49], and extracts of cocoa beans have been shown to inhibit the proliferation of A549 cells. Previous trials of antioxidant activity and MTT have determined which samples have the greatest potential for use in the analysis of cell cycle and apoptosis. To probe inhibition of cell growth mediated by RS and UWF extracts, we examined the cell cycle by flow cytometry. The effects of the RS and UWF extracts on cell cycle progression in A549 cells are shown in Table 1. After 48 h of growth, the population control in the sub-G_1_  G_0_/G_1_ phase reached values between 6.65 and 1.37% and between 75.75% and 75.80%, respectively. Meanwhile, cells treated in the sub-G_1_ population increased those treated in G_0_/G_1_ phase decreased in a dose-dependent manner in both treatments. The loss of the ability to regulate the cell cycle is characteristic of cancer cells and results in uncontrollable proliferation. The cell progression through the first gap phase of the cell cycle (G_1_) is a step which is frequently disordered in cancer [50].

Treatments with RS resulted in the highest percentage of cells in sub-G_1_ (77.01%, 10 mg/mL) and in G_0_/G_1_ (30.65%, 5 mg/mL) phases. As the G_0_/G_1_ phase population increased the G_2_/M phase population of cells decreased, whereas the population of phase S cells showed no difference within 48 h. Treatment with UWF cocoa at a concentration of 10 mg/mL showed the highest value for sub-G_1_ (50.27%) reduction within phases G_0_/G_1_ and S and an increase in the G_2_/M phase. Treatment with 5 mg/mL of UWF cocoa showed the smallest number of cells in sub-G_1_ and the G_0_/G_1_ phases and therefore a greater cell population in the G_2_/M phase (33.95%).

Cocoa-derived pentameric procyanidin (pentamer) caused a G_0_/G_1_ cell cycle arrest in human breast cancer MDA MB-231, MDA MB-436, MDA MB-468, SKBR-3, and MCF-7 cells and in benzo(a)pyrene-immortalized 184A1N4 and 184B5 cells, whereas normal human mammary epithelial cells in primary culture and spontaneously immortalized MCF-10A cells were resistant [51]. Similarly, procyanidin-enriched extracts from cocoa caused growth inhibition with blockade of the cell cycle at G_2_/M phase in human colonic Caco-2 cells [52], and EC induced S phase arrest in the cell cycle progression in LoVo colon cancer cells [53].

Our results indicate that cocoa nibs extracts were able to modify cell cycle. The deregulation in cell cycle control is a fundamental aspect in the development of cancer. Faults in the cell cycle regulation process can cause a greater proliferation of cancer cells. However, the reversal of this process leads to a delay in growth and induces cell death [54].

#### 3.2.3. Apoptosis

We examined the effect of RS and UWF cocoa on apoptotic death in A549 cells.  Table 2 shows the percentages of viable, early apoptotic, late apoptotic, and nonapoptotic cells treated with 5 mg/mL and 10 mg/mL RS and UWF cocoa.  Figure 4 shows the influence of the extracts of cocoa nibs on the rate of apoptosis. Cells treated with 5 mg/mL and 10 mg/mL RS and UWF cocoa for 48 hours resulted in a significant increase in the percentage of apoptotic cells compared with untreated cells (control). The concentrations used in this cellular model promoted a change in cell cycle and induction of apoptosis by lower doses than isolated compounds or cocoa products used in studies with human models. [55–57]. This has been demonstrated in a study by Ottaviani et al. [57], where adult males were given 1.5 mg/kg of a concentrated cocoa solution, and two hours after ingestion it was noted that there were many metabolites of flavonols in cocoa in the blood of the volunteers.

**Figure 5:**
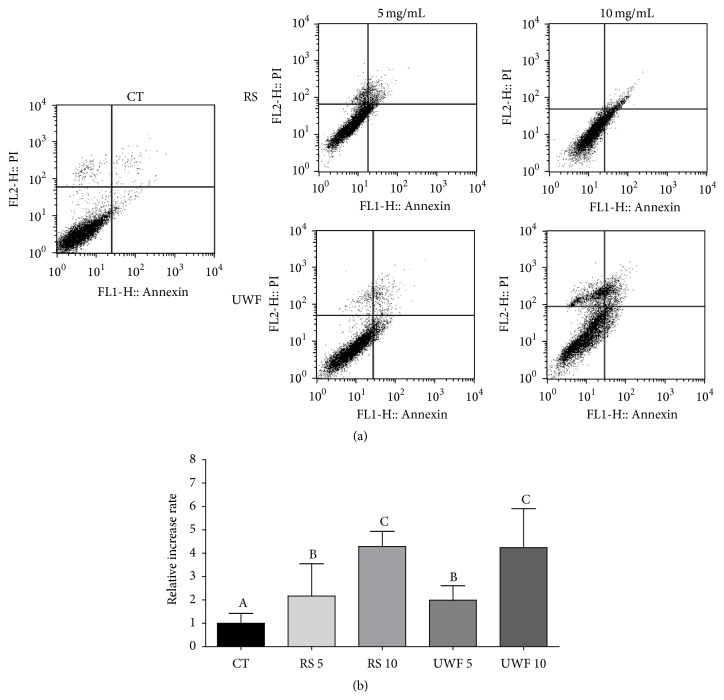
The effect of unroasted well fermented cocoa and roasted slates on the process of programmed death in A549 cells after treatment for 48 h. (a) Flow cytometry analysis of UWF cocoa and RS according to the exposure time and concentration of the compounds. (b) Quantitative effects of UWF cocoa and RS at 5 mg/mL and 10 mg/mL on A549 cells after exposure for 48 h. The results are expressed as mean ± SD, with significant differences between untreated cells (CT) and cells treated with UWF cocoa and RS (5–10 mg/mL) compared by 1-way ANOVA followed by Tukey's multiple comparison post hoc test. ^*∗*^ *p* < 0.05. ^*∗∗*^ *p* < 0.01.

Increase of early apoptosis was observed in A549 cells treated with 10 mg/mL RS, whereas increase in late apoptosis was observed in cells treated with 10 mg/mL UWF cocoa for 48 h. A549 cells incubated with 5 mg/mL and 10 mg/mL RS UQG for 48 hours showed a decrease in the population of viable cells and an increase of up to 4.3 times in the percentage of apoptotic cells compared with control, in a dose-dependent manner (Table 2 and Figure 5).

Apoptosis is characterized by a series of distinct changes in cell morphology, loss of cell attachment, cytoplasmic contraction, DNA fragmentation, and other biochemical changes, including the activation of caspases through extrinsic and/or intrinsic mitochondrial pathways [58]. Increased resistance to apoptosis is a hallmark of many tumor cells. The functional inhibition of specific antiapoptotic factors may provide a rational basis for the development of novel therapeutic strategies. Therefore, apoptotic deficiency is considered to be a major cause of therapeutic resistance of tumors, since many chemotherapeutic and radiotherapeutic agents act through the induction of apoptosis [59]. However, the apoptotic effect of the active ingredient of cocoa bean samples on A549 cells has not yet been studied in detail.

## 4. Conclusion

In conclusion, cocoa extract presented itself as a potent antioxidant agent, with antioxidant capability influenced by the processing method and extraction. Slate cocoa beans extract inhibited cell proliferation, arrested cell cycle in different phases, and increased apoptosis in human lung carcinoma cells, in a time-dependent and dose-dependent manner. Our study has far reaching health relevance as cocoa could be projected as functional foods which, in addition to providing nutrition, would provide preventive therapeutic value against the development of cancer.

## Figures and Tables

**Table 1:** Effect of extracts of cocoa, cocoa nibs roasted slates (RS), and unroasted well fermented (UWF) cocoa (5–10 mg/mL) on cell cycle progression in human lung cancer cell line after 48 hours.

	Cell cycle phase	CT	5 mg/mL	10 mg/mL
RS	sub-G_1_	6.65 ± 1.00	55.65 ± 0.49	77.10 ± 2.26
	G_0_/G_1_	75.75 ± 4.45	30.65 ± 0.92^*∗*^	20.63 ± 0.90^*∗*^
	S	5.40 ± 0.77	4.89 ± 0.16	0.74 ± 0.40^*∗*^
	G_2_/M	7.68 ± 1.77	6.73 ± 0.38	0.70 ± 0.64^*∗*^

UWF	sub-G_1_	1.37 ± 0.29	13.55 ± 2.62^*∗*^	50.28 ± 7.09^*∗∗*^
	G_0_/G_1_	75.80 ± 6.02	25.65 ± 7.57^*∗*^	21.78 ± 5.35^*∗*^
	S	5.61 ± 1.17	2.64 ± 1.58^*∗*^	2.20 ± 1.01^*∗*^
	G_2_/M	11.58 ± 3.54	33.95 ± 3.04^*∗∗*^	16.08 ± 4.01^*∗*^

Results are expressed as the percentage of total cells. The data represent mean ± SD values of triplicate experiments. Tukey's test; ^*∗*^ *p* < 0.05; ^*∗∗*^ *p* < 0.01.

**Table 2:** Effect of extracts of cocoa nibs roasted slates and unroasted well fermented cocoa (5–10 mg/mL) on programmed cell death in human lung cancer cell line after 48 hours.

Stages of cell death	CT	RS (mg/mL)		UWF (mg/mL)	
		5	10	5	10
Viable cells (Annexin V−/PI−)	95.80 ± 0.57	89.10 ± 2.26	86.73 ± 1.96	91.37 ± 1.33	72.03 ± 4.67^*∗*^
Early apoptosis (Annexin V+/PI−)	2.16 ± 1.03	1.80 ± 0.83	8.22 ± 2.48^*∗∗*^	3.87 ± 1.22	4.85 ± 2.05
Late apoptosis (Annexin V+/PI+)	0.90 ± 0.25	2.56 ± 1.30	4.92 ± 0.95^*∗*^	2.23 ± 0.67	8.13 ± 0.06^*∗∗*^
Nonapoptotic cells (Annexin V−/PI+)	1.12 ± 0.69	6.54 ± 0.13^*∗*^	0.14 ± 0.69	2.52 ± 0.68	14.93 ± 0.42^*∗∗*^

Results are expressed as percentage of total cells. The experiment is expressed as mean ± standard deviation; significant differences between untreated cells (CT) and cells treated with lycopene (5–10 *μ*M) were compared by one-way ANOVA with the posttest of Tukey (^*∗*^ *p* < 0.05; ^*∗∗*^ *p* < 0.01).
